# Nonalcoholic Fatty Liver Disease: An Emerging Modern-Day Risk Factor for Cardiovascular Disease

**DOI:** 10.7759/cureus.25495

**Published:** 2022-05-30

**Authors:** Gashaw Hassen, Abhishek Singh, Gizeshwork Belete, Nidhi Jain, Ivonne De la Hoz, Genesis P Camacho-Leon, Nitsuh K Dargie, Keila G Carrera, Tadesse Alemu, Sharan Jhaveri, Nebiyou Solomon

**Affiliations:** 1 Internal Medicine, University of Maryland Capital Region Medical Center, Largo, USA; 2 Medicine, Addis Ababa University, Addis Ababa, ETH; 3 Progressive Care, Mercy Medical Center, Baltimore, USA; 4 Medicine and Surgery, Parma University, Parma, ITA; 5 Internal Medicine, Saint Vincent Hospital, Worcester, USA; 6 Internal Medicine, Saint Agnes Hospital, Baltimore, USA; 7 Medicine and Surgery, Himalayan Institute of Medical Sciences, Dehradun, IND; 8 Hematology and Oncology, Brooklyn Cancer Care, Brooklyn, USA; 9 Internal Medicine, Sir Ganga Ram Hospital, New Delhi, IND; 10 Internal Medicine, Universidad del Zulia, Maracaibo, VEN; 11 Division of Research and Academic Affairs, Larkin Community Hospital, South Miami, USA; 12 División de Estudios Para Graduados, Universidad del Zulia, Facultad de Medicina, Maracaibo, VEN; 13 Family Medicine/Internal Medicine, Grand Canyon University, Arizona, USA; 14 Gastroenterology, Universidad de Oriente, Maturin, VEN; 15 Internal Medicine, Addis Ababa University, Addis Ababa, ETH; 16 Internal Medicine, Smt. Nathiba Hargovandas Lakhmichand Municipal Medical College, Ahmedabad, IND

**Keywords:** patient education, lifestyle modification, bariatric surgery, framingham risk score (frs), cardiovascular (cv) risk, cardiovascular disease (cvd), obesity, metabolic syndrome (mets), type 2 diabetes mellitus (t2dm), nonalcoholic fatty liver disease (nafld)

## Abstract

Nonalcoholic fatty liver disease (NAFLD), also named metabolic dysfunction-associated fatty liver disease (MAFLD), is a progressive disease spectrum encompassing simple steatosis, nonalcoholic steatohepatitis (NASH), fibrosis, and cirrhosis. It is a clinically silent disease leading to multiple extra-hepatic complications/comorbidities. It is an independent risk factor for cardiovascular disease (CVD), increasing susceptibility to hypertension, atherosclerosis, arrhythmia, myocardial dysfunction, cardiac valve deformation, and venous thrombosis through putative mechanisms including systemic inflammation, endothelial dysfunction, oxidative stress, insulin resistance, and altered lipid metabolism. Eventually, it increases the CVD prevalence, incident, and fatality, contributing to a huge health care burden. In fact, CVD is becoming the leading cause of mortality among patients with NAFLD. Other cardiometabolic risk factors coexisting with NAFLD may also accelerate the synergistic development of CVD, which warrants assessment targeting hypertension, diabetes mellitus (DM), obesity, and dyslipidemia to be an integral part of NAFLD care. Monitoring metabolic biomarkers (glucose, glycosylated hemoglobin [HbA1c], insulin, lipids, and lipoproteins), cardiovascular (CV) risk scores (American College of Cardiology/American Heart Association [ACC/AHA] or Framingham), and subclinical atherosclerosis (coronary artery calcification [CAC], carotid intima-media thickness [CIMT], and carotid plaque) are recommended for risk prediction and reduction. There is no universally accepted treatment for NAFLD, and lifestyle changes with weight loss of at least 10% are the mainstay of management. Combination therapy of ezetimibe and statins have a cardioprotective effect and help reduce liver fat.

Despite being an emerging risk factor for CVD and its rapidly increasing pattern affecting a quarter of the global population, NAFLD remains overlooked and undetected, unlike the other traditional risk factors. Hence, we conducted a comprehensive narrative review to shed more light on the importance of screening CVD in NAFLD patients. PubMed indexed relevant articles published from 2002 to 2022 (20 years) were searched in April 2022 using medical subject headings (MeSH) as "nonalcoholic fatty liver disease" [Mesh] AND "cardiovascular diseases" [Mesh]. Evidence from 40 observational studies, three clinical trials, one case series, 45 narrative reviews, four systematic reviews and meta-analyses, three systematic reviews, and one meta-analysis were summarized on the epidemiologic data, pathophysiologic mechanisms, clinical features, diagnostic modalities, overlapping management, perceived challenges and health literacy regarding the CVD risk attributed to NAFLD.

## Introduction and background

Nonalcoholic fatty liver disease (NAFLD) is a disease spectrum encompassing a continuum of progressive liver diseases, including simple steatosis (>5% of fatty infiltration of hepatocytes without injury), nonalcoholic steatohepatitis (NASH; fatty infiltration and inflammation), fibrosis (excessive deposition of extracellular matrix) and cirrhosis (advanced stage of fibrosis with distorted vasculature and architecture) [1-3].

NAFLD is diagnosed by excluding other causes of liver diseases such as alcohol and is considered as a hepatic manifestation of metabolic syndrome (MetS), which is characterized by three or more of the five conditions that increase the risk of heart disease, diabetes, stroke, and other health problems. These five conditions of the MetS that may occur together include high blood glucose, low blood high-density lipoprotein (HDL), high blood triglycerides, high blood pressure, and large waist circumference or "apple-shaped" body [2,4-5].

The pathogenesis of NAFLD involves a "multiple-hit" process including insulin resistance, oxidative stress, apoptosis, and adipokines with the first hit due to intrahepatic fatty acids (FA) accumulation which increases hepatocyte vulnerability to different secondary insults leading to inflammation and ultimate fibrosis [4,6]. A review by Buzzetti et al. best explained the "multiple hit" hypothesis for NAFLD pathogenesis as the cumulative effect of various insults, including insulin resistance, adipose tissue hormones, nutritional factors, gut microbiota, and genetic and epigenetic factors acting together [7].

Some of the well-established risk factors for NAFLD include obesity, type 2 diabetes mellitus (T2DM), dyslipidemia, MetS, and polycystic ovary syndrome (PCOS). Emerging conditions such as hypothyroidism, obstructive sleep apnea (OSA), hypopituitarism, hypogonadism, pancreaticoduodenal resection, and psoriasis are also found to have associations with NAFLD [8].

NAFLD is now the most common liver disease affecting a quarter of the world population, with the Middle East and South America having the highest regional prevalence while Africa is bearing the lowest burden [9]. Due to its stronger bidirectional association with MetS, NAFLD is more prevalent among high-risk groups such as patients with severe obesity undergoing bariatric surgery (>95%), T2DM (one-third to two-thirds), and dyslipidemia (half) contrary to normal-weight individuals (10%-15%) [9,10]. According to multiple studies, NAFLD affects 20% to 30% of the US population, with a higher prevalence among men, Hispanics, and older age [2,4]. 

NAFLD is mostly a silent disease with absent or subtle clinical manifestation, and it usually comes to medical attention as an incidental finding with elevated aminotransferase levels or radiographic features of fatty liver. Yet, a significant proportion of NAFLD patients have aminotransferase levels either typically normal or elevated by fewer times the upper normal limit [4].

Simple steatosis has a very good prognosis, but NASH can progress to cirrhosis and hepatocellular carcinoma (HCC) in 10% to 15% of cases [4]. Hence, NASH is becoming the rapidly increasing leading indication of liver transplantation in the US [2,11]. Though NAFLD primarily affects the liver, it is considered a multi-systemic disease associated with extrahepatic comorbidities/complications like cardiovascular disease (CVD) and chronic kidney disease (CKD) [10]. Sharing several cardiometabolic risk factors, NAFLD and CVD have strong associations [2]. Besides, various large population-based studies identified NAFLD as an independent risk factor for CVD [2,12]. To date, CVD is the most common cause of death (40%) in patients with NAFLD [13].

Despite the ongoing debate on the causal relationship between NAFLD and CVD, the established pathophysiological mechanisms linking the two include endothelial dysfunction, altered lipid metabolism, systemic inflammation, plaque formation/instability, oxidative stress, and insulin resistance [2,12]. The eventual disruption of the structure, function, and electrical activity of the cardiovascular system (CVS) then poses an increased risk for CVD, such as hypertension, atherosclerosis, arrhythmia, myocardial dysfunction, cardiac valve deformation, and venous thrombosis in NAFLD patients [12].

While the global pattern of NAFLD is dramatically changing in parallel with the increasingly sedentary lifestyle and unhealthy dietary choices, the emergence of NAFLD as the most common risk for CVD is largely overlooked [1,12]. Cardiovascular (CV) risk-assessment tools not incorporating NAFLD and the wider gap in health literacy among patients are a few of the multitude of challenges contributing to the overall burden of CVD attributed to NAFLD [13,14].

Aiming to emphasize the importance of screening CVD in NAFLD patients, a large body of evidence was retrieved and summarized based on the recent PubMed indexed publications spanning 20 years (2002-2022), which were searched in April 2022 using medical subject headings (MeSH) as "nonalcoholic fatty liver disease" [Mesh] AND "cardiovascular diseases" [Mesh]. Our review incorporated results from primary studies, including two case-control studies, 14 cohort studies, 24 cross-sectional studies, and three randomized control trials. Other sources of evidence our review relied on were three systematic reviews, one meta-analysis, four systematic reviews and meta-analyses, 45 prior review articles, one case series, two consensus statements from international experts, one discussion from a joint workshop, two patient pages from journals, four prominent websites from stakeholders, two guidances and one guideline. Relevant findings were used in the comprehensive review to discuss the epidemiologic, clinical, diagnostic, and interventional aspects of NAFLD in reference to its association with CVD. This review will also highlight the existing care-related gaps/challenges and the role of health education, particularly in addressing the overlapping management, such as lifestyle modification, to reduce the morbidity, mortality, and health care cost of both NAFLD and CVD.

## Review

Epidemiology of CVD in NAFLD patients

The burden of NAFLD is increasing rapidly in parallel with obesity, diabetes mellitus (DM), and MetS epidemics [8,15]. Most observational studies have also shown that NAFLD is independently associated with increased prevalence of CVD or CVD-related events, particularly in patients with metabolic dysfunctions, older age, and the general population [12].

A large meta-analysis by Wu et al. covering 165,000 participants from 34 studies conducted in different parts of the world demonstrated that NAFLD was associated with an increased risk of prevalent CVD (OR: 1.81; 95% CI: 1.23-2.66) and incident CVD (HR: 1.37; 95% CI: 1.10-1.72) [16]. In the same meta-analysis, NAFLD was associated with an increased risk of prevalent (OR: 1.87, 95% CI: 1.47-2.37) and incident (HR: 2.31, 95% CI: 1.46-3.65) coronary artery disease (CAD), prevalent (OR: 1.24, 95% CI: 1.14-1.36) and incident (HR: 1.16, 95% CI: 1.06-1.27) hypertension, and prevalent (OR: 1.32, 95% CI: 1.07-1.62) atherosclerosis [16].

A recent meta-analysis by Targher et al. based on 16 observational studies (nine prospective cohort studies, seven retrospective cohort studies) involving 34,043 patients with a median seven-year follow-up suggested that NAFLD patients had a 64% higher risk (OR: 1.64; 95% CI: 1.26-2.13) of developing fatal or non-fatal CV events in comparison to those without NAFLD [17].

NAFLD is an independent predictor for 10 years of CVD risk. Using the fatty liver index (FLI) in a Korean population study, a high Framingham 10-year CVD risk ≥10% was found in individuals with hepatic steatosis (FLI≥60) having OR: 2.56 (95% CI: 1.97-3.33) as compared to non-hepatic steatosis (FLI<30) after adjusting for confounders [18].

A Swedish cohort study of 229 participants having biopsy-proven NAFLD with a mean follow-up period of 26.4 years revealed that patients with NAFLD have an increased risk of mortality from CVD (HR: 1.55, CI: 1.11-2.15, p=0.01) and liver-related causes such as cirrhosis (HR: 3.2, CI: 1.05-9.81, p=0.041) and HCC (HR: 6.55, CI: 2.14-20.03, p=0.001) [19]. According to a US study on the national database of 2,826,531 deaths in 2018, heart disease mortality (10.33%) was identified as the second leading cause of death after liver-related mortality (45.83%) in patients with NAFLD [20]. Another multicenter retrospective investigation with a 10-year study period (2009-2018) which analyzed 10,071 hospitalization deaths aiming to explore NAFLD-related mortality in southern China, found that CVD mortality (35.6%) was ranked first, exceeding liver-related mortality (5.2%) which was ranked fifth [21].

Patients with increased liver fat were found to have higher incident CV risks such as hypertension (OR: 1.42; p<0.001) and T2DM (OR: 1.43; p<0.001) according to the analysis of 1051 Framingham Heart Study participants demonstrating bi-directional relationships between fatty liver and CVD risk factors among middle- to older-aged study participants who were followed for six years [22].

Despite some of the limitations like heterogeneity, residual confounding factors, selection bias at liver clinics, lack of clinical data on the reference population, and some missing cardiovascular events, most studies concluded that NAFLD is closely associated with adverse cardiovascular events.

Pathophysiologic mechanisms linking NAFLD with CVD

**Figure 1:**
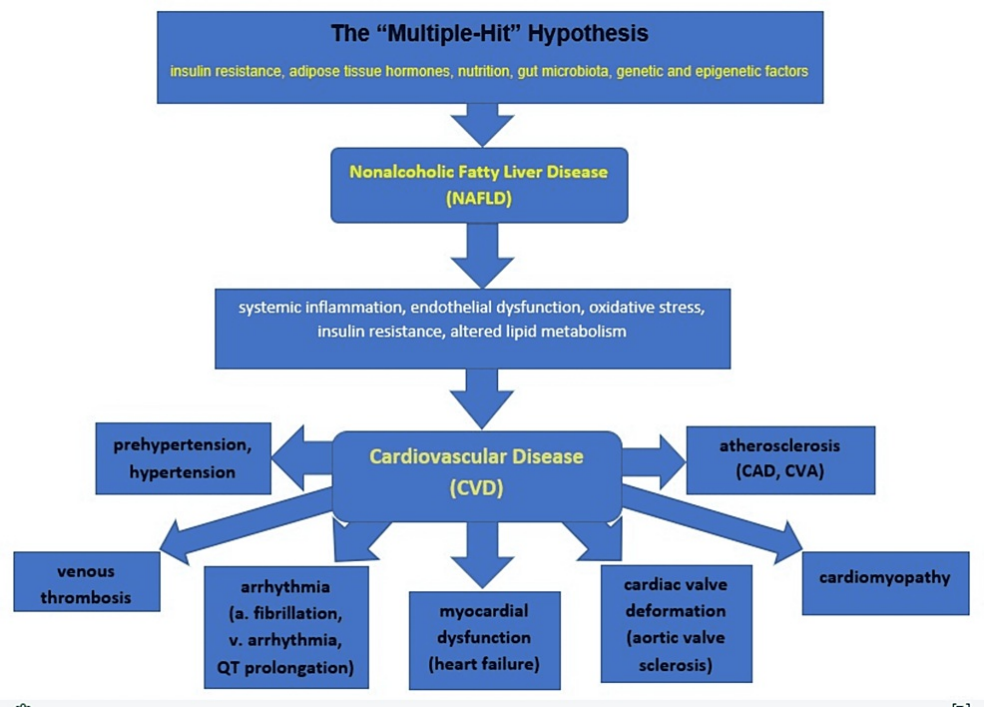
Summary of the pathophysiologic mechanisms behind NAFLD and its association with CVD NAFLD - nonalcoholic fatty liver disease; CVD - cardiovascular disease; a.fibrillation - atrial fibrillation; v. arrhythmia - ventricular arrhythmia; CAD - coronary artery disease; CVA - cerebrovascular accident Figure credit: Gashaw Hassen and Tadesse Alemu

Despite sharing risk factors through MetS, NAFLD and CVD are also linked through multiple pathophysiological mechanisms [23]. Some of the mechanisms by which NAFLD increases CVD risk include systemic inflammation, endothelial dysfunction, hepatic insulin resistance, oxidative stress, and altered lipid metabolism [2]. Experimental studies also demonstrated that NAFLD promotes CVD development through underlying mechanisms involving glucose and lipid dysregulation, imbalance of immunologic and neuroendocrine homeostasis, and activation of the prothrombotic system. Besides, metabolic risk factors intertwined with NAFLD may accelerate CVD development synergistically. Additional mechanisms contributing to CVD in patients with NAFLD include activation of the renin-angiotensin pathway, intestinal dysbiosis, and genetic and epigenetic factors [12]. NAFLD is considered an independent risk factor for CVD, but the causal association between the two is still not fully elucidated. Endothelial dysfunction, rapid pulse wave, coronary arterial calcifications, and carotid wall thickness are among the ultimate markers of CVD in NAFLD [24]. These complex pathophysiologic interactions subsequently predispose NAFLD patients to CVD risks, such as increased atherosclerosis, cardiomyopathy, and arrhythmia [2]. The following Figure 1 illustrates the summary of pathophysiologic mechanisms behind the association between NAFLD and CVD [2,12,23,24].

Clinical manifestations of CVD in NAFLD

NAFLD is a highly prevalent pathological disorder that is strongly linked to the clinical symptoms of MetS and is characterized by significant interpatient heterogeneity in severity and pace of liver disease development [25,26]. However, this liver-centric perspective ignores NAFLD's broader implications. Indeed, NAFLD is only one feature of a multisystem illness that causes much higher morbidity and mortality in people who are afflicted, with CVD being the leading cause of death, followed by extrahepatic malignancies and liver-related complications [19,27]. Given the tight links between NAFLD and the recognized CVD risk factors summarized by the MetS, such as abdominal obesity, hypertension, atherogenic dyslipidemia, and insulin resistance/dysglycemia, it's unsurprising that NAFLD is linked to an elevated risk of CVD [28-30].

Cardiovascular comorbidities in NAFLD patients are explained as follows.

NAFLD and Arterial Hypertension

The most frequent modifiable risk factor for CVD is arterial hypertension. According to current World Health Organization (WHO) estimates, high blood pressure (BP) causes around 54 percent of all strokes and 47 percent of all occurrences of ischemic heart disease [31-32].

Both prehypertension (PreHTN) and hypertension (HTN), irrespective of other cardiometabolic risk factors, are substantial risk factors for prevalent NAFLD. Controlled BP seems to be protective against NAFLD on its own, particularly in non-obese people. Controlled/no HTN also reduces the likelihood of moderate-to-severe hepatic fibrosis. Even in the absence of additional metabolic risk factors, BP in the PreHTN or HTN levels should trigger examination for NAFLD as part of a CVD risk assessment [33]. More research is needed to fully understand the function of BP regulation in the treatment of NAFLD, particularly in non-obese or lean people [33]. Finally, Ryoo et al. data, which came from a vast number of cohorts, revealed that NAFLD may be used as a clinical predictor of HTN. As a result, these data may provide long-term proof of NAFLD's causal role in HTN. Given the link between HTN and CVD, this research might be one of the clinical pieces of evidence supporting the link between NAFLD and CVD [34].

In prospective epidemiological studies conducted in France and Germany during nine and five-year observation periods, a 2-3 fold rise in the prevalence of arterial HTN was found. The incidence of incident HTN was linked to baseline gamma-glutamyltransferase (GGT) and fatty liver index (FLI), as well as a rise in GGT over time. Hepatic insulin resistance was shown to predict the development of HTN, suggesting that there is a relationship between liver indicators and HTN [35].

In their research, Lau et al. found that fatty liver disease, as defined by hepatic hyperechogenicity and elevated ALT levels, is linked to BP development and incident HTN. This link exists regardless of whether or not you drink alcohol. Individuals with liver hyperechogenicity and elevated ALT levels should have their BP tested on a regular basis, and lifestyle changes such as nutrition, physical exercise, and alcohol intake should be implemented. If ALT levels are high, ultrasonography should be performed, as it is done in normal treatment [36]. Increased liver brightness in ultrasonography is independently linked with greater circadian BP levels, according to Vasunta et al., and the connection is highest with systolic BP values [37]. To fully understand the mechanism, further research will be required.

Because NAFLD raises the risk of CVD, we recommend that people with fatty liver have their BP monitored closely. If BP medicine is required, choose one that has minimal metabolic side effects. The clinical value of 24-hour BP monitoring has yet to be determined; however, ambulatory BP measurement may be particularly useful for monitoring patients with fatty liver, based on the current data [37]. The 24-hour, daytime and night-time mean values of systolic or diastolic BP were significantly higher in people with hepatic steatosis on ultrasound, especially in contrast to those without NAFLD in the Oulu Project Elucidating Risk of Atherosclerosis (OPERA) study in Finland, while the association with non-dipping status showed only a trend (30.9 percent vs. 24.6; p=0.057) [37].

NAFLD and Coronary Artery Disease

The perivascular adipose tissue that covers the coronary arteries supports the development of a proinflammatory phenotype, and the accumulation of epicardial adipose tissue is linked to the prevalence, severity, and advancement of coronary artery disease (CAD), even when visceral obesity is not present [38]. Independent of established risk factors and MetS, there is evidence to suggest the link between NAFLD and subclinical atherosclerosis [2,12]. Future longitudinal research examining this link to determine causation with different ethnic groups included is needed [39]. A meta-analysis by Jaruvongvanich et al. found a substantial positive relationship between NAFLD and coronary artery calcium (CAC), supporting the significance of NAFLD as an independent predictor of CVD. CAC testing might be effective in identifying NAFLD patients who are at a greater risk of future cardiovascular events [40].

Haddad et al. meta-analysis suggests that NAFLD is linked to an elevated risk of clinical CVE, CAD, and stroke. To validate the link between NAFLD and CVE and demonstrate causation, well-designed large prospective epidemiologic studies across several demographic groups are required. Furthermore, it is unknown if active hepatitis, as demonstrated by increased serum transaminases, enhances the risk of CVE further than NAFLD diagnosis. In the meanwhile, it's a good idea to assess individuals with NAFLD for CV risk factors and occult CVD and start risk-reduction therapy (diet, statins, etc.) as soon as possible [41].

Nonalcoholic hepatic steatosis, as measured by the liver-to-spleen fat attenuation ratio estimated by multidetector computed tomography, is substantially connected with coronary artery plaques (particularly high-risk plaques), according to Osawa et al. meta-analysis. As a result, NAFLD might be a useful predictor of the acute coronary syndrome (ACS) [42].

According to Keskin et al., NAFLD and its severity may have an independent influence on both clinical outcomes (in-hospital and long-term) and CAD severity as measured by the SYNTAX score in patients with STEMI. The primary subgroup of these patients with grade 3 NAFLD exhibited greater rates of in-hospital death, stent thrombosis, and long-term mortality. The current study, together with earlier research, may point to the necessity of screening for NAFLD in STEMI patients [43].

NAFLD and Cardiac Arrhythmias

NAFLD is also linked to an increased risk of cardiac arrhythmias such as atrial fibrillation and ventricular arrhythmias, according to growing data [44]. Increasing data shows that NAFLD is closely linked to cardiac problems, including arrhythmias (i.e., AF, QTc prolongation, and ventricular arrhythmia), even when other CV risk factors and MetS symptoms are present. These results might explain, at least in part, why people with NAFLD have a higher risk of CV mortality​​ [45]. In their investigation, Käräjämäki et al. found significant epidemiological evidence that NAFLD is an independent risk factor for atrial fibrillation. Additional research is needed to show the pathophysiological mechanisms that underpin this link [46]. Mantovani et al. published the first research of its kind to show a positive and independent link between NAFLD and ventricular arrhythmias in type 2 diabetes patients who were sent for clinically recommended 24-hour Holter monitoring [45].

More extensive investigations are required to confirm these results and to better understand the underlying processes that cause this link [47]. In their research, Targher et al. were the first to show that ultrasound-diagnosed NAFLD is related to an increased incidence of AF in individuals with type 2 diabetes, even when other clinical risk factors for AF are taken into account [48]. The increased incidence of cardiac arrhythmias, particularly ventricular tachyarrhythmias, may further contribute to higher CVD morbidity and death among NAFLD patients.

It is worth mentioning that NAFLD exacerbates insulin resistance, predisposes to atherogenic dyslipidemia, and induces the production of proinflammatory, profibrogenic, and vasoactive mediators, all of which may contribute to cardiac and arrhythmic consequences [49].

NAFLD and Structural Heart Disease

There is mounting evidence that NAFLD harms not only the coronary arteries (promoting accelerated coronary atherosclerosis) but also all other anatomical structures of the heart, increasing the risk of cardiomyopathy (primarily left ventricular diastolic dysfunction and hypertrophy, resulting in the development of congestive heart failure), cardiac valvular calcification (primarily aortic-valve sclerosis), cardiac arrhythmias (primarily atrial fibrillation), and certain types of cardiac conduction defects [48,49]. All of these structural pathologies linked to NAFLD might contribute to the increased risk of CVD morbidity [48].

In a study conducted by Hallsworth et al., it was found that adults with NAFLD exhibited larger left ventricular walls in systole and diastole, as well as less longitudinal shortening than those without the disease. The NAFLD group had a greater eccentricity ratio, suggesting concentric remodeling. Peak whole-wall and endocardial strains were also greater in the NAFLD group. NAFLD did not affect cardiac metabolism as indicated by the phosphocreatine (PCr) to adenosine triphosphate (ATP) ratio. They concluded that, in the absence of metabolic alterations or overt cardiac illness, substantial changes in heart shape and function may be seen in people with NAFLD [50]. NAFLD is an independent predictor of cardiac calcification in both the aortic and mitral valves in individuals with type 2 diabetes, according to Mantovani et al. findings [51].

In conclusion, there is mounting evidence that metabolic dysregulation is the common denominator linking NAFLD to HTN, CAD, cardiac arrhythmias, and structural heart disease. It's no surprise that a group of specialists recently proposed the name "metabolic (dysfunction) associated fatty liver disease" (MAFLD) as a better overall phrase for NAFLD pathophysiology [52].

Diagnosis of NAFLD: non-invasive vs. invasive

Despite a better understanding of the disorder, diagnosing NAFLD remains a challenge, in part because patients are usually asymptomatic at early stages, and physicians must decide between non-invasive and invasive techniques to assess patients, the former being less reliable and the latter having a higher risk of complications and costs [53,54].

The association between NAFLD and MetS is so undeniable that NAFLD can be suspected in patients with MetS who have elevated liver enzymes with an aspartate transaminase/alanine transaminase (AST/ALT) ratio of less than 1. However, liver enzymes are normal in the majority of patients with confirmed NAFLD, which makes their diagnostic value questionable [53,55].

Non-invasive techniques such as imaging are typically used during the initial evaluation, with ultrasound being one of the most useful and recommended since it has a high sensitivity (60-94%) and specificity (66-98%) to diagnose steatohepatitis; it is readily available and inexpensive, but still is highly operator-dependent [54]. Typical findings include hyperechogenicity when compared to the renal cortex, areas of focal sparing, loss of delineation of the diaphragm, and poor definition of the intrahepatic architecture. However, its sensitivity decreases significantly when hepatic steatosis represents less than 30%, being this the main disadvantage. In addition, accuracy decreases in patients with renal disease or obesity, particularly if body mass index (BMI) is >40 kg/m² [54-56].

More sophisticated ultrasound techniques like the controlled attenuation parameter (CAP), which uses ultrasound with vibration-controlled elastography, are particularly helpful when the proportion of steatosis is smaller and represents a promising resource for earlier diagnosis with higher accuracy compared to conventional techniques [53]. In addition, endoscopic ultrasound (EUS) has been evaluated as an alternative diagnostic tool by Silva-Santisteban et al., who demonstrated that EUS could be especially helpful in obese patients when compared with regular ultrasound [57].

MRI is a highlyreliable method for diagnosing steatosis, and newer and more accurate techniques for diagnosing NAFLD have been developed based on MRI principles, such as MR spectroscopy (MRS), which measures the hepatic proton density fat fraction (PDFF) and allows for the detection of even mild cases of steatosis with high accuracy; however, it is not widely available, and it is expensive, which minimizes its feasibility [53,58].

Van Werben et al. developed a study where the effectiveness of ultrasound, CT, T1 weighted MR, and MR spectroscopy for the diagnosis of hepatosteatosis was compared. Results demonstrated that the MR-based methods had better performance and a stronger correlation with biopsy results (MRI with a sensitivity of 90% and specificity of 91% and MRS with a sensitivity of 91% and specificity of 87%) [59].

Serum biomarkers have also been used as non-invasive alternatives, and diagnostic algorithms have been developed, including the fatty liver index (FLI), hepatic steatosis index (HSI), lipid accumulation product (LAP), index of NASH, NAFLD liver fat score, among others (Table 1). The FLI was proposed in 2006 by Bedogni et al., which takes into consideration waist circumference, BMI, triglyceride, and gamma-glutamyl-transferase (GGT), with a sensitivity of 61% and specificity of 86% for the diagnosis of hepatosteatosis, representing an economical and practical option [54-56,60]. Huang et al. in a cross-sectional study with a sample of middle-aged and elderly Chinese patients found that the FLI had high sensitivity and specificity, 79.89% and 71.51%, respectively, for the diagnosis of NAFLD [61].

**Table 1:** Biomarkers used for non-invasive diagnostic algorithms GGT - gamma-glutamyl transferase; BMI - body mass index; AST - aspartate transaminase; ALT - alanine transaminase; TG - triglycerides; WC- waist circumference; WHR - waist-to-hip ratio; HOMA - homeostatic model assessment; MetS - metabolic syndrome; DM - diabetes mellitus; T2DM - type 2 diabetes mellitus; FSI - fasting serum insulin References: [61-62,65-67]

Index/score	Variables
Fatty liver index (FLI)	WC, BMI, TG, GGT
Hepatic steatosis index (HSI)	AST/ALT ratio, BMI, sex, DM
Lipid accumulation product (LAP)	WC, TG
Index of NASH (ION)	WHR, TG, ALT, HOMA, sex
NAFLD liver fat score (NAFLD-LFS)	MetS, T2DM, FSI, AST, AST/ALT ratio

On the other hand, the hepatic steatosis index takes into consideration four aspects: the AST/ALT ratio, BMI, sex, and the concomitant presence of diabetes. Lee et al. found that HSI had a sensitivity of 93.1% and specificity of 92.4% for the diagnosis of NAFLD [62]. Interestingly, Wang et al. studied the association of FLI and HSI scores with carotid atherosclerosis in diabetic patients, finding that higher scores were linked to greater carotid intima-media thickness, suggesting that these scores might be useful to monitor vascular disease progression [63].

Lipid accumulation product (LAP) correlates waist circumference with triglycerides; it represents an indirect measure of excessive accumulation of lipids. Dai et al. in a cross-sectional study demonstrated the high accuracy of LAP for the diagnosis and evaluation of the severity of NAFLD [64]. Given the pathophysiological similarities between NAFLD and CVD, Khan et al. studied the use of LAP to assess CV risk and surprisingly found that it was a better predictor of risk than BMI [65].

The index of NASH is calculated using the waist-to-hip ratio, triglycerides, ALT, sex, and the Homeostasis Model Assessment of Insulin Resistance (HOMA). Compared with the scores mentioned before, this one is capable of not only diagnosing NAFLD but also predicting when it has progressed to NASH [56,66]. The NAFLD liver fat score takes into consideration fasting serum insulin, AST, AST/ALT ratio, and the presence of MetS and/or type 2 diabetes, with a sensitivity of 84% and a specificity of 69% to predict NAFLD according to the results obtained in a study developed by Kotronen et al. [53,56,67,68].

Despite efforts to develop safer and more practical procedures, the gold standard for diagnosing and staging NAFLD remains liver biopsy. The histopathologic hallmark in NAFLD is steatosis, which must be present in at least >5% of the sample to make the diagnosis; it is usually macrovesicular and most prominent in zone three (perivenular). NASH is defined by steatosis associated with signs of hepatocyte injury such as ballooning and the presence of a mixed inflammatory infiltrate (lymphocytes, eosinophil, scattered neutrophils usually surrounding ballooning [satellitosis], scattered microgranulomas), Mallory-Denk bodies (eosinophilic intracytoplasmic inclusions), apoptotic or acidophil bodies, megamitochondria, glycogenated nuclei and hepatic siderosis [53,69-71].

However, the high costs, associated morbidity, the fact that a sample represents only a small percentage of the total liver volume, potentially leading to false negatives, as well as the influence of both radiologists' and pathologists' expertise on the results, severely limit its diagnostic value and overall utility. In addition, it is not feasible to rely on histopathology to monitor the progression of the disease long term, which makes it imperative to continue exploring other alternatives and develop better strategies for both diagnosis and monitoring [53,54,72].

Cardiovascular risk assessment and reduction

Screening Cardiovascular (CV) Risks in Patients with NAFLD 

NAFLD has shown an independent high-risk association for CVD, and several studies have demonstrated the association between ALT and increased CV mortality. Elevated GGT level reported as a marker for NAFLD also revealed the independent association and adverse CVE [73]. The mechanism in how NAFLD increases the risk of CVE is not well elucidated so far; however, a previous study showed that the histologic severity of NAFLD was linked with increased arterial stiffness and endothelial dysfunctions. Besides, the presence of the cytokines-inducing inflammatory process contributes to endothelial dysfunction [74]. 

NAFLD is a condition closely related to MetS. MetS is a spectrum of diseases that predisposes to atherosclerotic cardiovascular disease (ASCVD), T2DM, and CKD. MetS-related diseases share common risk factors such as insulin resistance, compensatory hyperinsulinemia, abdominal obesity, dyslipidemia, hypertension, a prothrombotic state, and a proinflammatory state. Dyslipidemic states further include overproduction of very-low-density lipoprotein (VLDL) and triglyceride (hypertriglyceridemia) [75]. 

Risk Factor: Hypertension 

Evidence has demonstrated that NAFLD may induce multiple systemic adverse effects such as inflammation, activation of the renin-angiotensin system (RAS)-sympathetic nervous system (SNS), and insulin resistance (IR), all contributing to hypertension development. Hence, NAFLD is incriminated for triggering a proinflammatory process changing the endothelial integrity of arteries resulting in hypertension. A study by Zhao et al.summarizing different longitudinal studies indicated that NAFLD is highly linked with HTN. A German study also concluded that patients with NAFLD have a three-fold higher risk of developing HTN. It is obvious that NAFLD is emerging as a stronger risk factor for HTN [76].

Risk Factor: Diabetes Mellitus

Type 2 diabetes mellitus (T2DM) has a higher bidirectional risk association with NAFLD. The link between them is synergic as T2DM predisposes to developing NASH and late risk of hepatocellular carcinoma (HCC). Evidence from many studies has shown that NAFLD leads to a two-fold increase in T2DM. NAFLD increases hepatic and peripheral insulin resistance facilitating atherogenic dyslipidemia and inducing proinflammatory cytokines that promote T2DM. It is recommended to screen NAFLD patients for T2DM [77,78]. 

Risk Factor: Obesity and Dyslipidemia

Obesity is linked to several comorbidities such as CVD, stroke, T2DM, HTN, hyperlipidemia, CKD, malignancies, and NAFLD. NAFLD is showing an increasing trend among obese patients every year. Obesity is found to play a significant role in the initial process of NAFLD, as well as in the progression to NASH. The imbalance in the metabolic function of adipocytes and hepatocytes with regard to the accumulation of excess energy and lipids such as triglycerides contribute to NAFLD in obesity. Eventual lipotoxicity may induce mitochondrial defects, endoplasmic reticulum stress, and oxidative stress. A study on the Italian population showed that obesity increases the risk of NAFLD by 4.6 fold [79].

On the other hand, genetic predisposition has a key role that can be explained by the genomic association of NAFLD in non-obese patients independent of diabetes and obesity [75].

CV Risk Stratification in Patients with NAFLD

NAFLD has a 2.2-fold increase in overall mortality mainly due to CVD [77]. To minimize CV morbidity and mortality, NAFLD cases should be evaluated for CV risk regularly, at least every 1-2 years. Obesity, dyslipidemia, HTN, DM, and MetS, as discussed above, are the well-known risk factors warranting priority in the risk assessment. The initial visit should test fasting glucose or glycosylated hemoglobin (HbA1c). CV risk quantification/estimation based on Framingham risk score (FRS) and atherosclerotic cardiovascular disease (ASCVD) risk score using American College of Cardiology/American Heart Association (ACC/AHA) guidelines is also applicable to NAFLD patients to predict CVD. It is recommended to evaluate markers of subclinical atherosclerosis such as coronary artery calcification (CAC), carotid intima-media thickness (CIMT), and carotid plaque using imaging techniques [73]. Lipoprotein and apolipoprotein are the other parameters worth evaluating. Large research on 18,151 patients by Wu et al. determined that apolipoprotein B (ApoB) and lipoprotein (a) are better predictors of disease progression rather than FRS alone. FRS might underestimate the risk prediction of CVD in NAFLD patients when comparing high-risk populations vs. those with FRS<10% or with mild steatosis [80].

CV Risk Reduction Measures in NAFLD

Currently, there is no approved treatment for NAFLD. Experimental studies have demonstrated that the bile acids are important factors interacting with Takeda G-protein coupled membrane receptor (TGR5) and farnesoid X receptor (FXR) receptors to regulate the key aspect of glucose and lipid metabolism in NAFLD, which may represent the future treatment targets [78]. A randomized control trial by Cho et al. concluded that combination therapy of ezetimibe and statins are both safe and significantly reduce liver fat in patients with NAFLD [81]. Statins also have a CV protective effect [73]. 

Overlapping management approaches

The risk of CVD increases in patients with NAFLD. It is therefore very crucial to target the pathophysiological mechanisms that ultimately contribute to the development of NAFLD and its complications. The preventive management of NAFLD patients is like that of the general population, which includes increased physical activity, smoking cessation, monitoring blood pressure and glucose, and eating a balanced diet [82].

Weight loss is one of the interventions that has most helped to reduce the prevalence of NAFLD. In fact, weight reduction is now considered the most effective intervention for both lean and obese patients affected by NAFLD [83]. In general, all interventions that can lead to weight loss contribute to the resolution of NAFLD. In particular, a weight loss of at least 10% has been shown to resolve steatohepatitis and regression of fibrosis in liver tissues [84].

Dietary changes can be heterogeneous, and not all diets have the same metabolic effect. However, Khodami et al. conducted a clinical trial revealing sugar-free diet can reduce the risk of steatosis and hepatic fibrosis in obese patients while decreasing inflammatory markers and triglyceride levels [85].

Pharmacological treatments such as stimulants, lipase inhibitors, and glucagon-like peptide 1 (GLP1) analogs, which are approved for the treatment of obesity, are helpful. Though these drugs do not act directly on the liver, they do contribute to weight loss and eventually benefit obese patients. On the other hand, vitamin E and pioglitazone have been shown to directly improve the histological manifestations of NAFLD [83].

Bariatric surgery is the most effective therapeutic option for weight loss, but there exists access inequality among obese patients [86]. Despite the popularity of bariatric surgery in the treatment of morbid obesity and its related comorbidities, including NAFLD, post-op complications such as acute liver failure necessitating liver transplantation due to associated protein malnutrition and bacterial overgrowth are limiting its use [87,88].

Finally, ultrasound imaging and laboratory tests are essential in demonstrating the progression of NAFLD and verifying the efficacy of the treatments used. Although there are no specific tests to diagnose or monitor NAFLD, in high-risk patients, aminotransferases and γ-glutamyl-transpeptidase levels should be checked every six months. Similarly, an ultrasound can detect steatohepatitis and compare it to the tests performed at the initial diagnosis of NAFLD. A liver biopsy can be used to perform a comparative analysis of NAFLD after intervention in comparison to the time of diagnosis and to determine if fibrosis exists. In patients who have risk factors that can lead to NAFLD progression, histological evaluation is the best monitoring option [89].

NAFLD care-related challenges and proposed recommendations

There are multiple gaps/challenges facing patients, providers, researchers, and stakeholders within the healthcare system which contribute to poor NAFLD outcomes [8,85,90-103]. Table 2 below summarizes them with proposed recommendations in order to bridge the gaps and address the challenges rationally.

**Table 2:** Summary of the current gaps/challenges and proposed recommendations regarding NAFLD care NAFLD - nonalcoholic fatty liver disease; CME - continuing medical education; WGO - World Gastroenterology Organization; AASLD - American Association for the Study of Liver Diseases; BMI - body mass index References: [8,85,90-103] Note: The recommendations are proposed based on feasibility and cost-effectiveness.

Challenges	Recommendations
Poor awareness and understanding among patients with NAFLD	Provide health education
Knowledge gap among care providers in identifying NAFLD	Provide comprehensive training/CME based on current practical guides, scientific studies, and notable publications
Lack of uniform NAFLD screening guidelines	Utilize sponsored guidelines and guidances: WGO, AASLD
Other comorbidities coexisting with NAFLD are straining the health care system	Prioritize cardiometabolic risk assessment and optimize risk reduction
Late NAFLD diagnosis after complications developed	Employ early screening using non-invasive tests
Ethical dilemmas posed for diagnosing NAFLD, which has no effective treatment	Involve multidisciplinary team
Little attention given for NAFLD by the global public health community in terms of strategies and policies	Establish a working relationship with stakeholders involved in liver health
Heavy reliance on elevated liver enzymes for screening, which may miss the early stage of NAFLD with normal results	Rely on complete clinical evaluation, cardiometabolic risk assessment, and supplementary imaging/ultrasonography
Pioglitazone and vitamin E associated side effects such as postmenopausal bone loss, prostate/bladder cancer, hemorrhagic stroke	Recommend pharmacotherapies for selective patient population with strict follow up and limited duration
Presence of NAFLD in non-obese patients with BMI<25 kg/m2 (“lean NAFLD”) posing diagnostic/screening challenges	Risk screening, initial workup using ultrasound, addressing other cardiometabolic risks and reduction strategies
The slow progression of NAFLD with relatively longer asymptomatic interval posing resource challenges in clinical trials designed to develop diagnostic and therapeutic strategies	Accelerate drug development/approval process, involve stakeholders to address gaps and unmet needs

Patient education to optimize health literacy about NAFLD 

Since lack of awareness and clear understanding from the patient's side contributes to poor NAFLD care, patient education materials (PEMs) written in easy to read and understand manner are helpful. These materials can be printed and provided to the patients or be shared as digital copies through various platforms such as email communications, social media outlets, mobile applications, and QR code access. 

PEMs are one way of achieving health literacy that promotes self-care and improves health outcomes [104]. Thus, key information with some advice delivered through PEMs is likely to achieve behavioral changes and bring a positive influence on patients in managing their own modifiable risk factors. 

Patients can also access online sources of information that are published by prominent organizations in the area of NAFLD care, such as the National Institute of Diabetes and Digestive and Kidney Diseases (NIH-NIDDK), Journal of American Medical Association (JAMA), American College of Gastroenterology (ACG), American Liver Foundation (ALF) and Journal of Hepatology (JHEP) [90,105-108].

**Figure 2:**
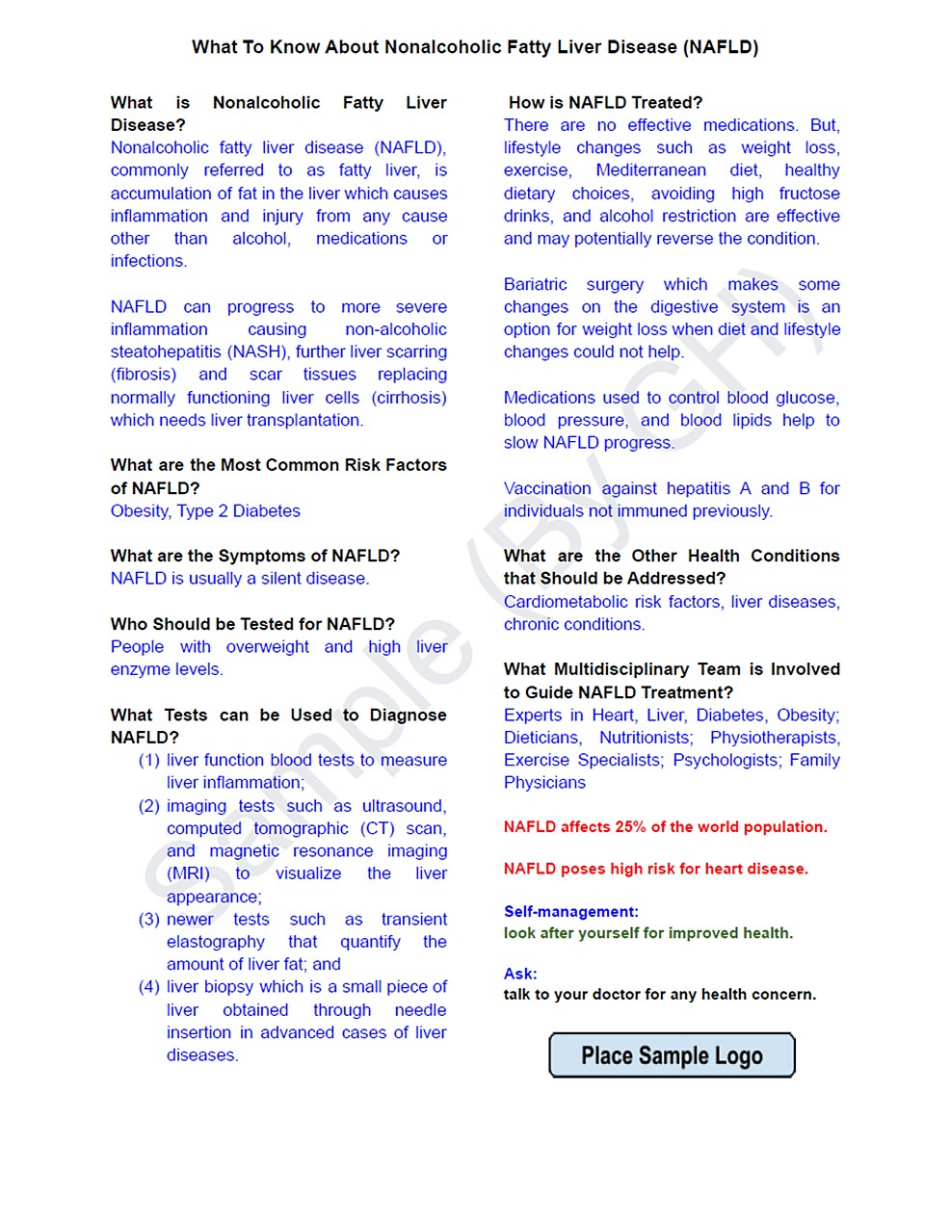
Sample patient education material (PEM) adapted and designed to create health awareness about NAFLD Placing the logo at the bottom of PEM can demonstrate the credibility of the source of information. Care providers can reproduce similar PEM by modifying the content and placing their own logo to suit the clinical settings. Credit: Gashaw Hassen

Besides, PEMs can be adapted, created, or translated to reflect topics that address the knowledge gaps and vital objectives for optimizing NAFLD care. Here is a sample PEM that highlights key pieces of information necessary for the public in general and NAFLD patients in particular as designed to create awareness based on health information mainly available on NIDDK and JAMA [104-105,109] (Figure 2). 

## Conclusions

Independent of traditional risk factors, NAFLD becomes an emerging predictor of CVD morbidity and mortality. Besides, it shares additional risks with other MetS-associated diseases such as T2DM multiplying the risk burden on CVD. NAFLD is both a diagnostic and therapeutic challenge. Employing non-invasive screening modalities helps for timely identification and intervention of both NAFLD and its comorbidities in high-risk patients. Assessment specific to cardiometabolic risk factors is very crucial for early risk stratification and reduction. The involvement of multi-disciplinary team members and stakeholders plays irrefutable roles in optimizing the management of NAFLD, its complications, and perceived barriers to care at various levels. Maximizing health literacy is beneficial to involve patients in self-care as a significant part of management for both NAFLD and CVD relies on lifestyle modification. Further investigation is paramount to identify the causal relationship between NAFLD and CVD, explore effective pharmacotherapy which can halt/reverse NAFLD progression, and develop uniform guidelines applicable in both primary and specialty clinical settings. 
